# Predicting treatment failure in stage III colon cancer patients after radical surgery

**DOI:** 10.3389/fonc.2024.1397468

**Published:** 2024-05-16

**Authors:** Hao Zeng, Xuejing Zhong, Wenxin Liu, Baofeng Liang, Xueyi Xue, Nong Yu, Dongbo Xu, Xiaojie Wang, Shuangming Lin

**Affiliations:** ^1^ Department of Gastroenterology and Anorectal Surgery, Longyan First Hospital, Fujian Medical University, Longyan, China; ^2^ Department of Science and Education, Longyan First Affiliated Hospital of Fujian Medical University, Longyan, China; ^3^ Department of Anaesthesia, Longyan First Hospital, Fujian Medical University, Longyan, China; ^4^ Department of Colorectal Surgery, Union Hospital, Fujian Medical University, Fuzhou, China

**Keywords:** stage III colon cancer, treatment failure, nomogram, Surveillance, Epidemiology, and End Results (SEER), TNM staging systems

## Abstract

**Purpose:**

The aim to assess treatment failure in patients with stage III colon cancer who underwent radical surgery and was analyzed using the nomogram.

**Methods:**

Clinical factors and survival outcomes for stage III colon cancer patients registered in the SEER database from 2018 to 2019 were analyzed, with patients split into training and testing cohorts (7:3 ratio). A total of 360 patients from the First Affiliated Hospital of Longyan served as an external validation cohort. Independent predictors of treatment failure were identified using logistic regression analyses. The nomograms was evaluated by concordance index (C-index), calibration curves, and the area under the curve (AUC), decision curve analysis (DCA) and clinical impact curves (CIC) assessed the clinical utility of nomograms versus TNM staging.

**Results:**

The study included 4,115 patients with stage III colon cancer. Multivariate logistic analysis age, tumor site, pT stage, pN stage, chemotherapy, pretreatment CEA levels, number of harvested lymph nodes, perineural invasion and marital status were identified as independent risk factors for treatment failure. The C-indices for the training and testing sets were 0.853 and 0.841. Validation by ROC and calibration curves confirmed the stability and reliability of the model. DCA showed that the net clinical effect of the histogram was superior to that of the TNM staging system, while CIC highlighted the potentially large clinical impact of the model.

**Conclusions:**

The developed Nomogram provides a powerful and accurate tool for clinicians to assess the risk of treatment failure after radical surgery in patients with stage III colon cancer.

## Introduction

Colorectal cancer (CRC), accounting for an estimated 1.9 million new cases and 935,000 deaths globally in 2020, is recognized as the third most prevalent type of cancer and the second leading cause of cancer-related mortality. It represents approximately one in every ten cancer cases and deaths (1). Stage III colon cancer, characterized by lymph node metastasis, often involves deep infiltration of the colon wall and adjacent lymph nodes, markedly diminishing survival rates (2). Despite substantial progress in the clinical management of stage III colon cancer over recent decades (3), accurately predicting treatment outcomes for individual patients remains a formidable challenge (4). Consequently, devising precise predictive tools to ascertain a patient’s risk of treatment failure post-radical surgery is essential for enhancing treatment outcomes.

Treatment failure, typically defined as any recurrence or death a patient experiences within 12 months post-surgery, signifies a grave clinical outcome with a dire prognosis (5–7). This not only severely impacts patient survival and quality of life but also exerts a significant influence on healthcare resource allocation and the formulation of treatment strategies. Various factors contribute to treatment failure, including pathological characteristics, treatment decisions, and individual patient variances. Research conducted by Giammauro Berardi (7) and others has firmly established that factors such as the primary tumor site, T-stage, lymph node status, disease-free interval, and the quantity and dimensions of metastatic foci are intimately linked with the treatment failure of colorectal cancer liver metastases.

Acknowledging the absence of validated instruments for predicting treatment failure risk in stage III colon cancer patients, this study is committed to a thorough examination and identification of pivotal risk factors leading to treatment failure in patients post-radical surgery for stage III colon cancer. Moreover, we will assess the efficacy of the newly developed column-line diagram in predicting treatment failure and juxtapose it with the prevailing TNM staging system. This endeavor will empower clinicians to pinpoint the risk of treatment failure in stage III colon cancer patients with greater precision, thereby refining treatment approaches and curtailing the incidence of treatment failure.

## Materials and methods

### Included participants

This retrospective cohort study utilized data from patients diagnosed with stage III colon cancer (limited to those with a single primary tumor) extracted from a total of 18 registries using the National Cancer Institute’s SEER Cancer database for the period 2018 to 2019. Data screening and retrieval were conducted using SEER*Stat 8.4.2 software (http://seer.cancer.gov/seerstat/). Eligible patients were selected based on the following inclusion criteria: (1) diagnosis of stage III colon cancer according to the International Classification of Diseases for Oncology, Third Edition (ICD-O-3); (2) availability of active follow-up data with well-defined causes of mortality for deceased patients. Exclusion criteria encompassed patients with non-primary tumors, unclear pathological diagnoses, less than the 12-month follow-up, appendiceal tumors or ambiguous tumor locations, unclear pathological grades, unspecified tumor sizes, uncertain numbers of harvested lymph nodes, or unclear tumor grades as per the AJCC classification (8th version). For each patient, the study collected the following information: age, sex, race, tumor stage, histological grade, tumor site, tumor size, number of harvested lymph nodes, scope of regional lymph nodes, marital status, pretreatment carcinoembryonic antigen (CEA) levels, receipt of chemotherapy, perineural invasion (PNI), receipt of postoperative chemotherapy/radiation, presence of tumor deposits, survival time in months, and survival status.

### Data extraction

**Figure 1:**
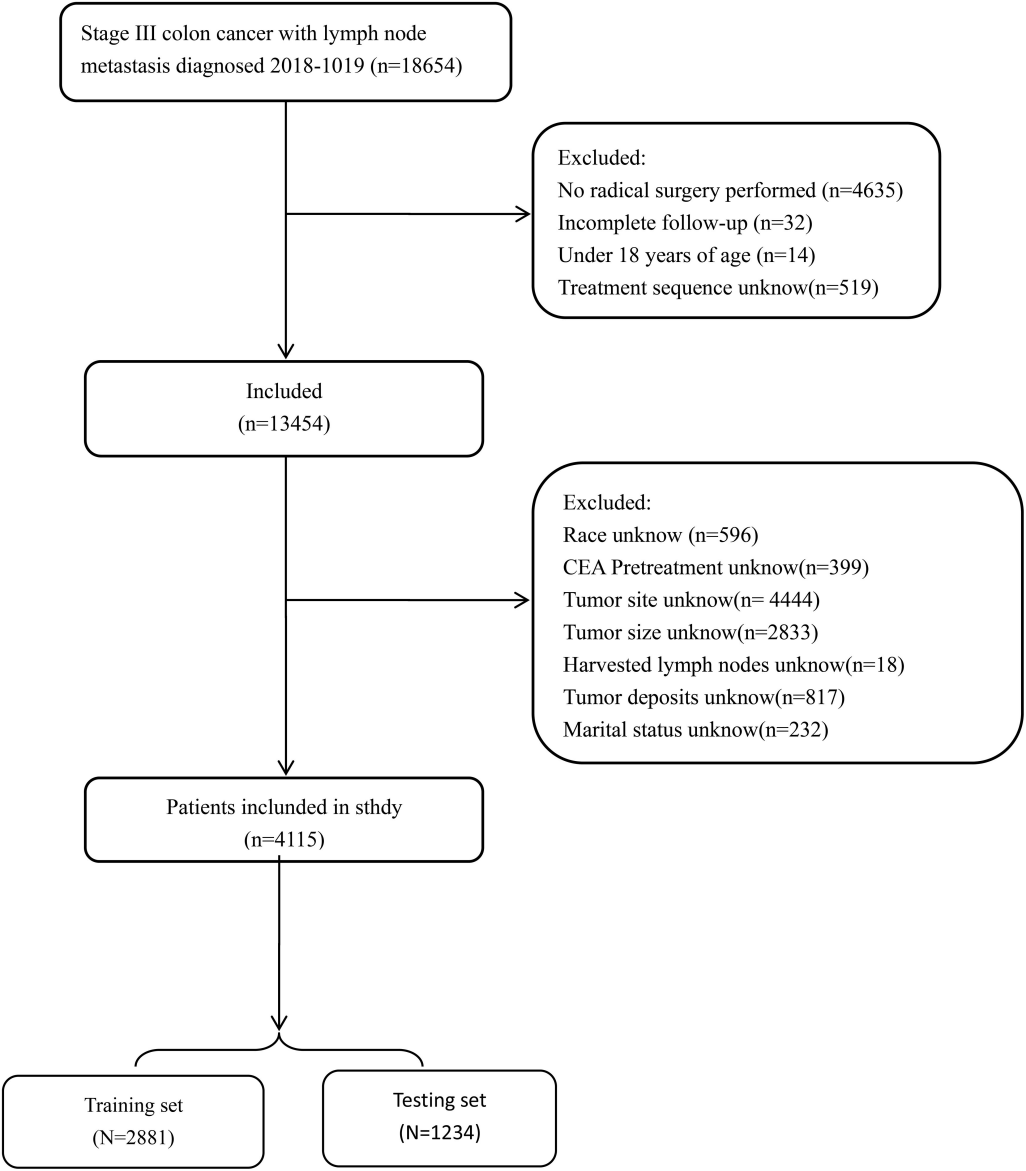
Flowchart of patient cohort definition.

Patients were divided into training and testing cohorts at a ratio of 7:3. The training set consisted of a total of 2,881 patients, while the testing set comprised 1,234 patients (**Figure 1**). Marital status was regrouped as married or unmarried (single, widowed, divorced and separated). The number of lymph nodes (nLN) sampled was regrouped as <12 or ≥12, and tumor size was regrouped as < 5 cm or ≥5 cm according to the X-tile program (8). A total of 360 patients with stage III colon cancer was collected from Longyan First Affiliated hospital of Fujian Medical University to validate model externally. This study was conducted in line with the Declaration of Helsinki and approved by the Ethics Committee of Longyan First Affiliated hospital of Fujian Medical University (number: LYREC2024-k027-01).

### Statistical analysis

All the patients were randomly assigned to the training and testing cohorts using a ratio of 7:3. The primary outcome was treatment failure, defined as any recurrence or death within 12 months from surgery (6, 7). The categorical variables were expressed as numbers and percentages (n,%), and the differences in the distribution of the variables between the training and validation cohorts were assessed using Pearson’s chi-square test. Univariate logistic regression analysis was performed on the training cohorts to identify the risk factors for treatment failure. The significant risk factors were included in the multivariate logistic regression analysis to identify the independent risk factors. The performance of the nomogram in the training and validation cohorts was evaluated as follows. The concordance index (C-index) was used to evaluate the nomogram’s predictive performance, and a calibration curve with a 1000-times bootstrapping was plotted to evaluate the consistency between the actual and predicted probabilities. The area under the curve (AUC) with the 95% confidence interval (CI) of a receiver operating characteristic (ROC) curve was calculated to evaluate the discrimination ability of the nomogram. An area under the roc curve (AUC) value above 0.7 was considered to have good predictive capabilities. Finally, a decision curve analysis (DCA) was performed to compare the clinical utility of the nomogram and standard AJCC TNM staging system. All statistical analyzes were carried out using the R software (version 4.3.1), and a two-sided p-value below 0.05 was deemed statistically significant.

## Results

### Basic characteristics of the patients

The demographic and clinical characteristics of stage III colon cancer patients in both the training and testing cohorts are summarized in **Table 1**. A total of 4,115 stage III colon cancer patients were enrolled in the study, of whom 50.5% (n = 2,078) were males, and the rest were females (49.5%, n = 2037). Most patients were white (77.5%, n = 3191) and aged above 60 years (70.6%, n = 2904). With the exception of histology and lymph node ratio no significant differences in demographic and clinical characteristics were observed between the training and testing groups.

**Table 1:** Baseline characteristics.

	Overall population (N=4115)	Training set (N=2881)	Testing set (N=1234)	P-value
Age				
<50 years	482(11.7%)	332 (11.5%)	150 (12.2%)	0.719
50-59 years	729(17.7%)	521 (18.1%)	208 (16.9%)	
60-69 years	1016(24.7%)	714 (24.8%)	302 (24.5%)	
70-79 years	991(24.1%)	699 (24.3%)	292 (23.7%)	
80+ years	897(21.8%)	615 (21.3%)	282 (22.9%)	
Sex				
Female	2037(49.5%)	1425 (49.5%)	612 (49.6%)	0.965
Male	2078(50.5%)	1456 (50.5%)	622 (50.4%)	
Race				
American Indian/Alaska Native	46(1.1%)	34 (1.2%)	12 (1.0%)	0.46
Asian or Pactific Islander	394(9.6%)	287 (10.0%)	107 (8.7%)	
Black	484(11.8%)	344 (11.9%)	140 (11.3%)	
White	3191(77.5%)	2216 (76.9%)	975 (79.0%)	
Site				
Ascending Colon	811(19.7%)	578 (20.1%)	233 (18.9%)	0.083
Cecum	928(22.6%)	617 (21.4%)	311 (25.2%)	
Descending Colon	240(5.8%)	164 (5.7%)	76 (6.2%)	
Hepatic Flexure	188(4.6%)	132 (4.6%)	56 (4.5%)	
Rectosigmoid Junction	318(7.7%)	219 (7.6%)	99 (8.0%)	
Sigmoid Colon	1089(26.5%)	798 (27.7%)	291 (23.6%)	
Splenic Flexure	124(3%)	84 (2.9%)	40 (3.2%)	
Transverse Colon	417(10.1%)	289 (10.0%)	128 (10.4%)	
Histopathology				
Adenocarcinoma	3744(91.0%)	2648 (91.9%)	1096 (88.8%)	0.006
Mucinous adenocarcinoma	318(7.7%)	201 (7.0%)	117 (9.5%)	
Signet ring cell carcinoma	53(1.3%)	32 (1.1%)	21 (1.7%)	
pT				
T1	153(3.7%)	110 (3.8%)	43 (3.5%)	0.369
T2	381(9.3%)	265 (9.2%)	116 (9.4%)	
T3	2546(61.9%)	1805 (62.7%)	741 (60.0%)	
T4a	742(18.0%)	498 (17.3%)	244 (19.8%)	
T4b	293(7.1%)	203 (7.0%)	90 (7.3%)	
pN				
N1a	1318(32.0%)	948 (32.9%)	370 (30.0%)	0.27
N1b	1262(30.7%)	871 (30.2%)	391 (31.7%)	
N1c	283(6.9%)	199 (6.9%)	84 (6.8%)	
N2a	705(17.1%)	496 (17.2%)	209 (16.9%)	
N2b	547(13.3%)	367 (12.7%)	180 (14.6%)	
Surgical procedure				
Hemicolectomy	2270(55.2%)	1573 (54.6%)	697 (56.5%)	0.538
Partial colectomy	1697(41.2%)	1203 (41.8%)	494 (40.0%)	
Total colectomy	148(3.6%)	105 (3.6%)	43 (3.5%)	
Scope of regional lymph nodes				
1 to 3 regional lymph nodes	42(1.0%)	28 (1.0%)	14 (1.1%)	0.884
4 or more regional lymph nodes	4018(97.7%)	2814 (97.7%)	1204 (97.6%)	
None	55(1.3%)	39 (1.4%)	16 (1.3%)	
Radiation				
None/Unknown	4033(98.0%)	2829 (98.2%)	1204 (97.6%)	0.232
Yes	82(2%)	52 (1.8%)	30 (2.4%)	
Chemotherapy				
None/Unknown	1444(35.1%)	998 (34.6%)	446 (36.1%)	0.374
Yes	2671(64.9%)	1883 (65.4%)	788 (63.9%)	
CEA pretreatment				
CEA negative/normal	1485(36.1%)	1053 (36.5%)	432 (35.0%)	0.307
CEA positive/elevated	1193(29.0%)	815 (28.3%)	378 (30.6%)	
Unknown	1437(34.9%)	1013 (35.2%)	424 (34.4%)	
Harvested lymph nodes				
<12	229(5.6%)	157 (5.4%)	72 (5.8%)	0.675
≥12	3886(94.4%)	2724 (94.6%)	1162 (94.2%)	
Lymph node ratio				
<0.05	707(17.2%)	524 (18.2%)	183 (14.8%)	0.031
>0.2	1183(28.7%)	814 (28.3%)	369 (29.9%)	
0.05 to <0.2	2225(54.1%)	1543 (53.6%)	682 (55.3%)	
Tumor deposits				
No	3047(74.0%)	2146 (74.5%)	901 (73.0%)	0.343
Yes	1068(26.0%)	735 (25.5%)	333 (27.0%)	
Tumor size				
<5cm	2226(54.1%)	1567 (54.4%)	659 (53.4%)	0.584
≥5cm	1889(45.9%)	1314 (45.6%)	575 (46.6%)	
Perineural invasion				
No/Unknown	3293(80.0%)	2296 (79.7%)	997 (80.8%)	0.444
Yes	822(20.0%)	585 (20.3%)	237 (19.2%)	
Marital status				
Married	2190(53.2%)	1551 (53.8%)	639 (51.8%)	0.24
Unmarried	1925(46.8%)	1330 (46.2%)	595 (48.2%)	
Income				
$35,000 - $49,999	494(12.0%)	344 (11.9%)	150 (12.2%)	0.962
$50,000 - $74,999	2081(50.6%)	1463 (50.8%)	618 (50.1%)	
$75,000+	1449(35.2%)	1012 (35.1%)	437 (35.4%)	
< $35,000	91(2.2%)	62 (2.2%)	29 (2.4%)	

### Risk factors for treatment failure

Univariate logistic regression analysis revealed associations between age, tumor site, histology, pT stage, pN stage, surgical procedure, radiation, chemotherapy, pretreatment CEA levels, number of harvested lymph nodes, presence of tumor deposits, Lymph node ratio, perineural invasion and marital status with treatment failure. Subsequently, in the multivariate logistic analysis age, tumor site, pT stage, pN stage, chemotherapy, pretreatment CEA levels, number of harvested lymph nodes, perineural invasion and marital status were identified as independent risk factors for treatment failure (**Table 2**).

**Table 2:** Univariate and multivariable analysis of risk factors for treatment failure.

Variable	Univariate analysis				Multivariate analysis			
	HR	95% CI		P-value	HR	95% CI		P-value
Age								
<50 years	Reference				Reference			
50-59 years	2.19	1.03	4.68	0.042	1.94	0.87	4.32	0.106
60-69 years	4.85	2.41	9.77	<0.001	4.16	1.98	8.74	<0.001
70-79 years	9.89	4.98	19.65	<0.001	7.25	3.49	15.04	<0.001
80+ years	26.11	13.17	51.61	<0.001	9.85	4.73	20.48	<0.001
Sex								
Female	Reference							
Male	0.87	0.72	1.05	0.154				
Race								
American Indian/Alaska Native	Reference							
Asian or Pactific Islander	0.61	0.23	1.57	0.304				
Black	1.09	0.43	2.73	0.859				
White	1.12	0.46	2.73	0.797				
Site								
Ascending Colon	Reference				Reference			
Cecum	1.05	0.80	1.38	0.711	0.72	0.52	1.00	0.048
Descending Colon	0.71	0.45	1.12	0.137	0.76	0.44	1.31	0.316
Hepatic Flexure	0.88	0.55	1.41	0.595	0.90	0.52	1.55	0.699
Rectosigmoid Junction	0.36	0.22	0.60	<0.001	0.57	0.31	1.08	0.083
Sigmoid Colon	0.53	0.40	0.71	<0.001	0.86	0.57	1.29	0.461
Splenic Flexure	1.05	0.61	1.81	0.865	1.22	0.62	2.39	0.559
Transverse Colon	1.21	0.87	1.68	0.265	1.00	0.67	1.50	0.990
Histopathology								
Adenocarcinoma	Reference				Reference			
Mucinous adenocarcinoma	1.49	1.06	2.08	0.021	1.10	0.73	1.23	0.550
Signet ring cell carcinoma	3.15	1.55	6.43	0.002	2.27	0.93	2.47	0.376
pT								
T1	Reference				Reference			
T2	1.28	0.60	2.71	0.525	1.27	0.56	2.90	0.569
T3	1.95	1.00	3.77	0.048	1.52	0.73	3.15	0.260
T4a	3.76	1.90	7.41	<0.001	2.48	1.16	5.30	0.019
T4b	4.57	2.24	9.34	<0.001	3.65	1.61	8.25	0.002
pN								
N1a	Reference				Reference			
N1b	1.16	0.91	1.49	0.236	1.25	0.91	1.72	0.174
N1c	1.42	0.96	2.09	0.080	0.78	0.46	1.35	0.382
N2a	1.35	1.02	1.80	0.039	1.14	0.72	1.81	0.575
N2b	2.28	1.71	3.04	<0.001	1.96	1.13	3.38	0.016
Surgical procedure								
Hemicolectomy	Reference				Reference			
Partial colectomy	0.65	0.53	0.80	<0.001	0.91	0.67	1.23	0.550
Total colectomy	0.99	0.61	1.60	0.961	1.32	0.71	2.42	0.376
Scope of regional lymph nodes								
1 to 3 regional lymph nodes	Reference							
4 or more regional lymph nodes	0.56	0.25	1.28	0.170				
None	0.86	0.29	2.56	0.790				
Radiation								
None/Unknown	Reference				Reference			
Yes	0.08	0.01	0.61	0.014	0.19	0.02	1.53	0.118
Chemotherapy								
None/Unknown	Reference				Reference			
Yes	0.10	0.08	0.12	<0.001	0.15	0.12	0.20	<0.001
CEA Pretreatment								
CEA negative/normal	Reference				Reference			
CEA positive/elevated	1.92	1.50	2.47	<0.001	1.36	1.01	1.83	0.045
Unknown	2.23	1.76	2.82	<0.001	1.37	1.04	1.81	0.027
Harvested lymph nodes								
<12	Reference				Reference			
≥12	0.35	0.25	0.49	<0.001	0.42	0.27	0.66	<0.001
Lymph node ratio								
<0.05	Reference				Reference			
>0.2	2.02	1.51	2.69	<0.001	1.41	0.80	2.48	0.240
0.05 to <0.2	1.11	0.84	1.46	0.475	1.10	0.74	1.65	0.631
Tumor deposits								
No	Reference				Reference			
Yes	1.43	1.16	1.76	<0.001	1.21	0.92	1.60	0.182
Tumor size								
<5cm	Reference				Reference			
≥5cm	1.70	1.41	2.06	<0.001	1.26	1.00	1.60	0.053
Perineural invasion								
No/Unknown	Reference				Reference			
Yes	1.44	1.16	1.80	0.001	1.49	1.13	1.98	0.005
Marital status								
Married	Reference				Reference			
Unmarried	1.81	1.50	2.19	<0.001	1.27	1.02	1.60	0.036
Income								
$35,000 - $49,999	Reference							
$50,000 - $74,999	0.85	0.64	1.14	0.289				
$75,000+	0.80	0.59	1.09	0.157				
< $35,000	1.21	0.64	2.28	0.565				

### Construction of the nomogram

**Figure 2:**
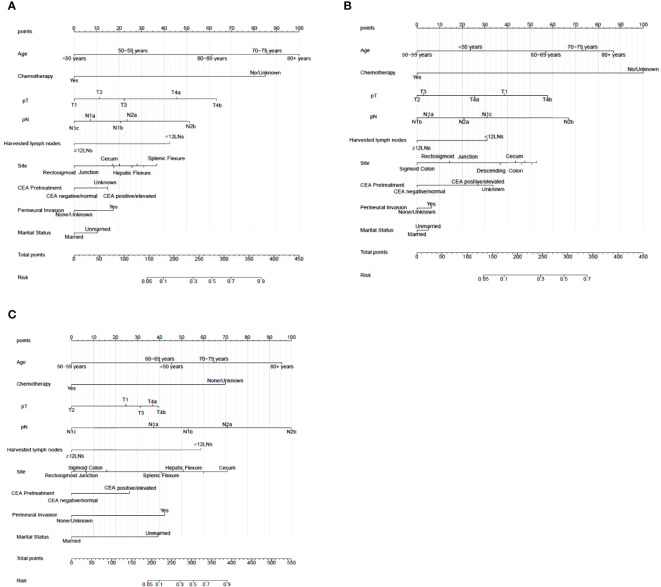
Nomogram for treatment failure of stage III colon cancer patients in training cohort, testing cohort and validation cohort. **(A)** Nomogram in training cohort. **(B)** Nomogram in testing cohort. **(C)** Nomogram in validation cohort. To estimate the risk of treatment failure, the point of each variable was calculated by drawing a straight line from the patient variable value to the axis marked “points.” The total points are converted to the “Risk” on the lowest axis.

**Figure 3:**
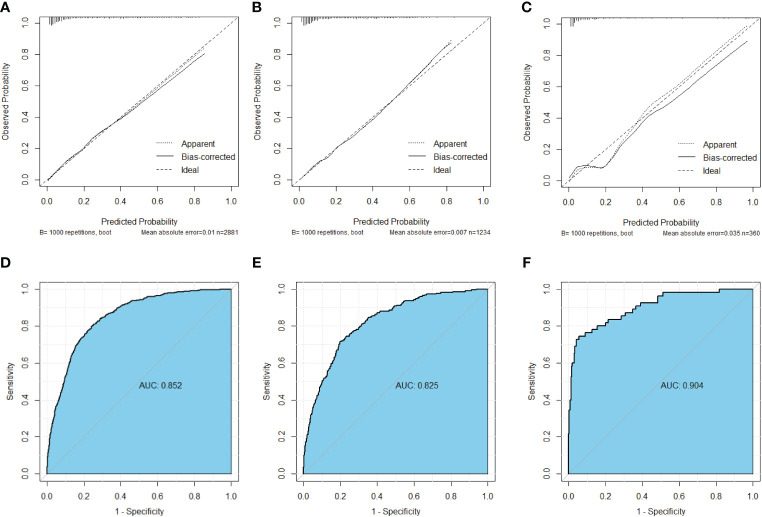
Calibration curves of nomograms for treatment failure. **(A)** Calibration curve in the training cohort. **(B)** Calibration curve in the testing cohort **(C)** Calibration curve in the validation cohort. **(D)** ROC curve in the training cohort. **(E)** ROC curve in the testing cohort. **(F)** ROC curve in the validation cohort.

**Figure 4:**
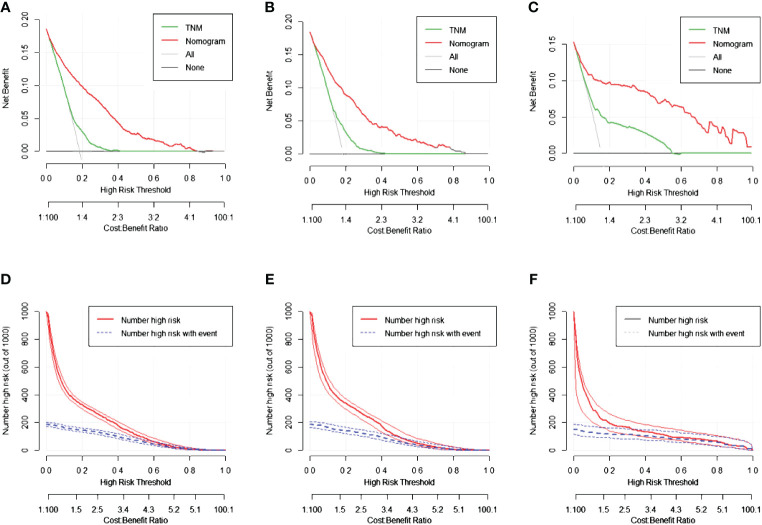
The decision curve analysis (DCA) curves and clinical impact curve (CIC) curves of nomogram for treatment failure, the nomograms (red line) had a better clinical net value than the TNM staging system (green line). **(A)** DCA curve in the training cohort. **(B)** DCA curve in the testing cohort. **(C)** DCA curve in the validation cohort. **(D)** CIC curve in the training cohort. **(E)** CIC curve in the testing cohort. **(F)** CIC curve in the validation cohort.

Nomograms were constructed using independent risk factors for treatment failure after radical surgery in patients with stage III colon cancer, as shown in **Figures 2A–C**. These predictive maps provide scores corresponding to each risk factor, with the total score representing the sum of all variable scores. The risk of treatment failure was determined by drawing a line from the total score to the corresponding risk score. In the training cohort, the nomogram C-index was 0.853, and in the testing cohort, the nomogram C-index was 0.841. Following the validation cohort, the C-index for the treatment failure nomogram was 0.904, and these results suggest that the nomogram model has strong predictive performance and reliability. As shown in the calibration curves, the nomograms show a very good match between the predicted and observed results in both the training and testing cohorts, with the prediction curves being very similar to the diagonal (**Figures 3A–C**). In validation cohort, the nomograms showed slightly poorer predictions, with AUC values of 0.852 and 0.825 for the nomograms in the training and testing cohorts, respectively. In validation cohort, the AUC value was 0.904 (**Figures 3D–F**). Decision curve analysis in each cohort showed that the nomogram achieved better net benefit in predicting treatment failure in each cohort compared to the TNM AJCC colon cancer staging system (**Figures 4A–C**). The solid line represents the number of people at high risk of treatment failure according to our model, and the dashed line represents the number of people who actually failed treatment in the CIC. (**Figures 4D–F**).

## Discussion

Stage III colon cancer is defined as a tumor that has invaded adjacent tissues and spread to one to three regional lymph nodes but has not yet developed distant metastases (9). Treatment at this stage usually involves a multidisciplinary combination of surgical, radiotherapy, and chemotherapy approaches. Although these therapeutic strategies have improved survival rates, the prognosis of patients remains uncertain (10). Studies have shown that higher T-stage, regional lymph node involvement, perineural infiltration, and high-stage tumor outgrowth are independently associated with disease recurrence, cancer-related deaths, and reduced overall survival (OS) (4, 11). Additionally, genetic factors such as KRAS, NRAS, and BRAF mutations, along with microsatellite instability (MSI) status, influence the behavior of tumors and their response to certain treatments, although their association with prognosis may vary from individual to individual (12). Surgery is considered the primary treatment option for stage III colon cancer (13). However, patients with stage III colon cancer are more prone to postoperative complications and tumor recurrence, which can lead to treatment failure, as they often require more extensive surgery and a range of postoperative treatments (14). Therefore, there is a need to identify factors associated with treatment failure in stage III colon cancer in order to optimize the treatment of these patients.

In this study, we extracted clinical data from the SEER database on 4,386 patients with stage III colon cancer. Univariate and multivariate logistic regression analyses were performed on these patients, identifying several predictive risk factors for treatment failure: advanced age, tumor location in the splenic flexure, a high TNM stage, absence of chemotherapy, positive or elevated preoperative carcinoembryonic antigen (CEA) levels, obtaining fewer than 12 lymph nodes, presence of neural invasion, and unmarried status. Interestingly, mortality in the first year post-surgery exceeded 50% for patients older than 80 years, with a one-month postoperative mortality rate of 31%. This heightened mortality rate, particularly in patients over 80 years of age, is primarily attributed to cardiopulmonary complications. Additionally, our analysis revealed a higher incidence of cancer-unrelated deaths in the older patient group, whereas cancer-specific mortality remained similar between the two age groups.

Perioperative supportive measures, including aggressive respiratory support, are crucial to preventing pneumonia and should be strongly encouraged (15). In several population-based studies, a higher T-stage was significantly correlated with a decrease in 5-year OS rates, with T3 tumors at 87.5% and T4 tumors at 71.5%, which further decreased to 46% for T4b tumors (16). Elevated preoperative CEA levels have been identified as an independent prognostic factor for stage I-III colorectal cancer (CRC) following radical resection. For patients with lymph node-negative CRC and preoperative CEA levels > 10 ng/ml, intensive follow-up or adjuvant chemotherapy is advisable (17). Adjuvant chemotherapy (ACT) is critical in enhancing survival rates post-radical surgery for patients with stage II-III CRC (18). In stage III colorectal cancer, the incidence of perineural invasion (PNI) can reach up to 30%, marking PNI as an independent predictor of poor prognosis and decreased survival (11). Moreover, in a nationwide randomized clinical trial involving stage III colon cancer patients, social factors such as being divorced, separated, or widowed, and living arrangements significantly impacted patient outcomes. An increasing trend has been observed in Americans reporting nearly three times more frequently than in the past that they lack confidants for discussing serious matters (19).

Tumor location in the splenic flexure is identified as a high-risk factor for treatment failure, attributed to the variable and incompletely understood lymphatic drainage in this region (20). Tumors located at the left flexure often exhibit stenosis, infiltration beyond the plasma membrane, and a higher incidence of mucinous histology, leading to a more frequent recurrence as peritoneal carcinomatosis (21). Furthermore, our validation data indicate that tumor location in the cecum is an independent risk factor for treatment failure. This may be due to several reasons: patients with right colon cancer have significantly lower 5-year overall survival rates than those with left colon cancer. Additionally, the quality and/or extent of mesenteric resection may hold particular importance in the treatment of right-sided colon cancer, where the 5-year cancer-specific survival (CSS) post-recurrence is notably shorter in patients with right colon cancer compared to those with left colon cancer (22). In this study, we found that different tumor locations in stage III CRC patients have varied prognostic significance on recurrence and overall mortality following radical resection. This variability in prognostic factors may be attributed to the embryonic origin of the normal tissues from which these tumors develop, with the right hemi-colon deriving from the midgut and the left hemi-colon and rectum originating from the hindgut of the embryo (23). These differences in embryonic origins are reflected in the distinct genetic pathways of carcinogenesis (24). However, data regarding tumors located in the cecum are scarce, leading to potential bias due to the predominance of patients presenting with treatment failure.

The presence of tumor deposits (TDs) has been debated as a risk factor for treatment failure. Xuzhi Zheng et al. concluded that TDs are an independent, negative prognostic factor for both the 5-year OS and the 5-year CSS of stage III CRC patients. They suggested that TDs count should be considered in the prognosis evaluation of patients with N2 stage disease, with higher TDs counts (≥5) indicating a worse prognosis (25). Conversely, Hongjiang Pu et al. found that the prognosis of patients classified as N1c—indicating no lymph node metastasis but the presence of tumor deposits—is comparable to that of lymph node-positive patients without tumor deposition. They recommended adjuvant chemotherapy for patients with N1c colorectal cancer due to their high risk of recurrence and poor prognosis (26).

Our findings indicate that the choice of surgical procedure does not significantly impact the risk of treatment failure in stage III colon cancer patients. The surgical strategy for colon cancer, especially regarding colon resection, remains a subject of considerable debate. The optimal extent of bowel resection and lymph node dissection for colon cancer treatment is widely contested, with recommendations varying between hemicolectomy or extended hemicolectomy for tumors in the same or adjacent segments (27). Wang et al. (20) observed no significant differences in anastomotic dehiscence, reoperation rates, or mortality between patients undergoing left hemicolectomy and those receiving partial colectomy. Furthermore, Zeng Hao et al. emphasized the importance of a comprehensive evaluation of the patient’s pathological features, disease stage, and overall health status when considering D2 and D3 lymph node dissection (28). This suggests a need for personalized surgical planning based on individual patient factors rather than a one-size-fits-all approach.

There are some limitations of this study, the data were extracted retrospectively from the SEER database and may be biased due to lack of quality control of the included data. This limitation may affect the reliability of our findings. In addition, due to the lack of specific details in the SEER database, we were unable to study the effect of other potential risk factors on stage III colon cancer such as ASA, Performance Status or other clinical score, nutritional status, detailed chemotherapy regimen and perioperative information. In addition, the reliance on a single database for data collection limits the generalizability of our findings. To address this issue, we need to conduct further studies in multiple centers to validate the applicability of our findings and ensure their wider relevance.

## Data availability statement

The raw data supporting the conclusions of this article will be made available by the authors, without undue reservation.

## Ethics statement

The studies involving humans were approved by the Ethics Committee of Longyan First Affiliated Hospital of Fujian Medical University. The studies were conducted in accordance with the local legislation and institutional requirements. The ethics committee/institutional review board waived the requirement of written informed consent for participation from the participants or the participants’ legal guardians/next of kin because 1) This is an observational study and the researcher will not directly intervene or change the behavior or environment of the participants. Data for the study is derived from publicly available information or collected without interfering with the normal activities of the participants, therefore there is no direct risk to the participants. 2) All data collected will be de-identified to ensure that participants cannot be identified directly or indirectly. In addition, the reporting or publication of research results will be based solely on the results of data analysis, and no personal information will be disclosed. 3) The project will be supervised by an ethical review committee to ensure that the research process complies with ethical standards. Any changes in research activities will be resubmitted for review. Study has no greater than minimal risk to subjects.

## Author contributions

HZ: Writing – original draft, Visualization, Software, Methodology, Investigation, Formal Analysis, Data curation. XZ: Writing – original draft, Methodology, Funding acquisition, Formal Analysis. WL: Writing – original draft, Methodology, Data curation. BL: Writing – original draft, Visualization, Validation, Resources. XX: Writing – original draft, Software, Methodology, Investigation. NY: Writing – original draft, Validation, Software, Data curation. DX: Writing – review & editing, Project administration, Conceptualization. XW: Writing – review & editing, Supervision, Project administration, Conceptualization. SL: Writing – review & editing, Supervision, Project administration, Funding acquisition, Conceptualization.
